# 

**Published:** 1852-01

**Authors:** 


### CONTENTS OF NO. I.

### ORIGINAL COMMUNICATIONS.

### ESSAYS AND CASES.

ART. I.—On the Use of Large Doses of Opium in the Treatment of Fever, Inflammation, and Dysentery. By ANSON G. HENRY, M. D., of Springfield, Illinois. - 1

ART. II.—Two Cases of Puerperal Peritonitis. Reported by J. M. LAWRENCE, M. D., of Nashville, Tennessee; late resident Pupil at Bellevue Hospital, New York. - 12

ART. III.—A Case of Puerperal Convulsions relieved by Blood-letting. By BENJ. C. DRURY, M. D., of Breckinridge County, Ky. - - - - - - - 15

ART. IV.—A Case of Ununited Fracture of the Tibia, of Four Years Standing successfully treated. By W. G. WILLIAMS, M. D., of Chillicothe, Ohio. - - - - 16

ART. V.—An Essay on the Sore-Mouth peculiar to Nursing Females; read before the New Albany Medical Society. By DR. P. S. SHIELDS, of New Albany, Ia. - - 21

### REVIEWS.

ART. VI.—Outlines of Chemistry for the Use of Students. By WILLIAM GREGORY, M. D., Professor of Chemistry in the University of Edinburgh. First American, from the Second London edition. Revised, corrected, and enlarged by J. Milton Sanders, M. D., L. L. D., Professor of Chemistry, Pharmacy, and Toxicology, in the E. Medical Institute of Cincinnati; and Professor of Natural Philosophy and Chemistry in the Literary Department of the Memphis Institute, etc - - - - - 29

ART. VII.—Operative Surgery, based on Normal and Pathological Anatomy. By J. F. MALGAIGNE, Professor Agregé de la Faculté de Medicine de Paris, Chirurgien de l' Hopital de Lourcine, Chevalier de la Legion d' Honneur, et du Mente Militaire de Pologne, etc., etc. Translated from the French, by FREDERICK BRITTAN, A. B., M. D., M. R. C. S. L. - - - - - 34

ART. VIII.—Elements of Physiology, including Physiological Anatomy. By WILLIAM B. CARPENTER, M. D., F. R. S., F. G. S., Examiner in Physiology and Comparative Anatomy in the University of London; &c., &c. - - - 37

ART. IX.—Cox's Companion to the Sea Medicine Chest, and Compendium of Domestic Medicine; particularly adapted for Captains of Merchant Vessels, &c., with plain rules for taking the medicines; directions for restoring suspended animation, the method of obviating the effect of poisons, &c., &c. Revised and considerably enlarged by R. DAVIS, Member of the Royal College of Surgeons. - 53

ART. X.—The Pocket Formulary and Synopsis of the British and Foreign Pharmacopœias, comprising standard and approved formulæ for the preparations and compounds employed in Medical Practice. By H. BEASLEY, M. D. 54

ART. XI.—Intermarriage; or the mode in which, and the causes why, Beauty, Health, and Intellect, result from unions, and Deformity, Disease, and Insanity, from others: illustrated by delineations of the Structure and Forms, and descriptions of Functions and Capacities, which each parent, in any pair, bestows on Children—in conforming with certain natural laws, and by an account of corresponding effects in the Breeding of Animals. With eight illustrative drawings. By ALEXANDER WALKER. - 55
ART. XII.—Essays on Infant Therapeutics. To which are added, observations on Ergot; history of the origin of the use of mercury in inflammatory complaints; together with the statistics of the deaths from poisoning in New York, in the years 1841—'2—'3. By JOHN B. BECK, M. D., Professor of Materia Medica and Medical Jurisprudence in the College of Physicians and Surgeons of the University of New York; &c. &c. - - 57
ART. XIV.—The Elements of Materia Medica and Therapeutics. By JONATHAN PEREIRA, M, D., F. R. S. and L. S.—Third American edition, enlarged and improved by the author. Including notices of the mechanical substances in use in the civilized world, and forming an Encyclopedia of Materia Medica. Edited by Joseph Carson, M. D., Professor of Materia Medica and Pharmacy in the University of Pennsylvania. - - - - - 59

SELECTIONS FROM FOREIGN AND HOME JOURNALS.

Muscular Contraction—Cadaveric Rigidity—French and American Experiments, &c. - - - - - 61
Unconsolidated Fracture of the Thigh successfully treated by Acupuncture. - - - - - 70
A Bilobed, cr Sacculated Bladder. - - - - - 73
Chloride of Sodium as a Substitute for Sulphate of Quinine, in Intermittent Fever. - - - - - 76
Medical Properties and Uses of the Cimicifuga Racemosa. - 77
Cod Liver Oil in Phthisis. - - - - - 81
Compression of the Aorta in Uterine Hemorrhage. - - 82
Case of Inversion of the Vagina coming on during Labor. - 83
Study of Anatomy in Egypt. - - - - - - 84

ORIGINAL INTELLIGENCE.

Our Journal, - - - - - - 85
Inanition, - - - - - - - 86
Heat of Vegetables and Animals, - - - - - 87
Dr. Dowler's Physiological Researches, - - - 89
Death by Lightning, - - - - - - 89
Life Insurance Explained, - - - - - - 90
Practical Chemistry, - - - - - 91
Meteorological Table, for the month of December, 1851. - 92

